# Aristotelian universals, strong immanence, and construction

**DOI:** 10.1007/s11229-023-04421-x

**Published:** 2024-01-19

**Authors:** Damiano Costa, Alessandro Giordani

**Affiliations:** 1 Institute of Philosophy, Università Della Svizzera Italiana, Lugano, Switzerland; 2 Department of Philosophy, Università Cattolica di Milano, Milan, Italy

**Keywords:** Universals, Grounding, Immanence, Dependence, States of affairs

## Abstract

The Aristotelian view of universals, according to which each universal generically depends for its existence on its instantiations, has recently come under attack by a series of ground-theoretic arguments. The last such arguments, presented by Raven, promises to offer several significant improvements over its predecessors, such as avoiding commitment to the transitivity of ground and offering new reasons for the metaphysical priority of universals over their instantiations. In this paper, we argue that Raven's argument does not effectively avoid said commitment and that Raven's new reasons fail. Moreover, we present a novel ground-theoretic interpretation of the Aristotelian view, referred to as strong immanence, and introduce a new argument against the Aristotelian view, intended to sidestep any commitment to the transitivity of ground.

The Aristotelian view of universals, according to which each universal generically depends for its existence on its instantiations, has recently come under attack by a series of ground-theoretic arguments (Alvarado, 2020; Costa, 2021; Raven, 2022; replies in Giordani & Tremolanti, 2022; Imaguire, 2021). Such arguments seem all to build on alleged circularities of priority, ground, or dependence, generated by the Aristotelian view. Indeed, on the one hand, the Aristotelian view requires (1) universals to depend on their instantiations, while, on the other hand, there seem to be powerful reasons to claim the reverse is true too: (2) instantiations depend on the universals they are instantiations of. The last such arguments, presented by Raven (2022), promises to offer two significant improvements over its predecessors. First, it does not assume, and is therefore free from controversies over, the transitivity of grounding (Raven, 2022, p. 7). Second, it offers new reasons in favour of (2), based on two popular views on how states of affairs are constructed from their constituents.

The aim of this paper is to conduct a comprehensive assessment of the problems prompted by Raven's paper. Specifically, we undertake the following four tasks: (i) show that Raven's reasons for supporting (2) fail; (ii) show that Raven's argument is not successful in avoiding commitment to the transitivity of grounding; (iii) present a novel ground-theoretic interpretation of the Aristotelian view, referred to as strong immanence; (iv) introduce a new argument against the Aristotelian view, intended to sidestep any commitment to transitivity. Throughout our analysis, we delve into several interesting questions that emerge from Raven's work, particularly those concerning the interplay between grounding, mereology, and non-mereological constitution.

## Raven’s argument

Raven’s argument against the Aristotelian view can be summarized in three basic steps. First, Raven argues that the Aristotelian view implies the metaphysical priority of instantiations over universals. Second, Raven offers independent reasons for the metaphysical priority of universals over their instantiations. Third, he claims that the conjunction of first two claims violates the asymmetry of metaphysical priority.

As to the first step, Raven briefly reviews three ways of characterizing the Aristotelian view, namely in terms of location (i.e. universals are spatiotemporally located), mereology (i.e. universals are parts of particulars) and of the principle of instantiation (i.e. universals must be instantiated in order to exist), and discards each of them. As a result, he claims that the best way, if not the only viable one, to properly characterize the Aristotelian view is in terms of grounding, which he, following nowadays standard practice, takes to be a “metaphysically determinative explanation” which he assumes to be asymmetric (Raven, 2022, p. 2).^1^ The Aristotelian view is then nicely captured along the lines of a principle he calls (Raven, 2022, p. 4)

immanence: If a universal U^2^ exists, then some instantiation of it helps ground U’s existence.

In other words, the Aristotelian view has it that the existence of a universal is at least partially grounded in the obtaining state of affairs that something instantiates it. This provides the advertised metaphysical priority of instantiations over universals which constitutes step one of Raven’s argument.

The second step of Raven’s argument consists in providing reasons in favour of the opposite claim, namely the metaphysical priority of universals over their instantiations. Raven’s reasons are based on the explicit assumption that instantiations are states of affairs, and that universals help construct the states of affairs in which they are somehow involved. Raven distinguishes two accounts according to which universals help construct the states of affairs in which they are somehow involved, both of which display a characteristic Armstrongian flavour. According to the first one, universals construct states of affairs by way of non-mereological constituency (Armstrong, 1989, 1997), while according to the second one, universals construct states of affairs by way of mereologically overlapping with particulars (Armstrong, 2004a, 2004b). Raven drafts three arguments to the effect that either account implies the metaphysical priority of universals over their instantiations. More precisely, either view is argued to imply a principle he calls (Raven, 2022, p. 5)

construction: If a state of affairs [*U*p] obtains, then the existence of universal U helps ground [*U*p]’s obtaining.

The third step of Raven’s argument consists in arguing that immanence and construction entail a violation of the asymmetry of metaphysical grounding. At first sight, one might think that the violation is obvious. After all, immanence claims the priority of instantiations over universals, while construction claims the priority of universals over instantiations. However, on closer inspection, Raven subtly remarks that while immanence is generic, in that it claims that a universal is grounded in *some* of its instantiations (which is an existential generalization), construction is specific, in that it claims that a specific instantiation is partly grounded in the existence of a specific universal. Raven suggests that a violation of asymmetry can be anyway established if one assumes a principle of grounding he calls

existential grounding: When an item exists and has a feature, then its having that feature helps ground something’s having that feature.

In other words, pace Fine (2012), existential generalizations are grounded in their specific instances. So, if both existential grounding and immanence are true, Raven claims, the existence of a specific universal will be grounded not only in it’s being generically instantiated, but also in each *specific* instantiation of it—such as the existence of U which will be grounded in [*U*p]’s obtaining. Hence, the asymmetry of grounding is violated and Raven’s argument against the Aristotelian view is completed.

Raven’s argument can certainly be resisted in a number of ways. For example, as granted by Raven himself, both existential grounding and the asymmetry of grounding are not free from controversies. However, our aim here will be to focus on the two specific ways in which Raven’s argument allegedly improves upon its predecessors, such as Costa’s (2021) and Alvarado’s (2020). First, as mentioned above, Raven offers specific reasons for the priority of universals over their instantiations based on the assumption that universals help construct states of affairs. As such, Raven’s argument has not among its assumptions Costa’s (2021) Relata First Principle, according to which the existence of relata helps ground the obtaining of relational states of affairs – a principle which has come under attack in the recent literature (Giordani & Tremolanti, 2022; Imaguire, 2021). Second, as remarked by Raven himself (2022, p. 7), unlike Costa’s (2021), his argument does not assume the *transitivity of grounding*, and is therefore immune from arguments against it.

In what follows, we argue that both of Raven’s improvements fail: Raven’s specific reasons for the priority of universals over their instantiations fail (Sect. 2) and, appearances notwithstanding, Raven’s argument requires to assume the transitivity of grounding in order to be valid (Sect. 3). We don’t take this to be problematic, insofar as we believe grounding to obey transitivity. Still, on behalf of those who don’t take grounding to be transitive, we will also provide a new argument which is indeed free from any commitment to transitivity, thus somehow vindicating Raven’s intuition that this could be done (Sect. 4).

## Raven’s argument for the priority of universals over states of affairs

The first alleged improvement of Raven’s argument over its predecessors consists in the specific reasons it provides for the priority of universals over their instantiations. We shall now have a closer look at such reasons. The upshot is going to be that such reasons fail in several important respects.

More precisely, Raven captures the alleged priority of universals over their instantiations by means of the already mentioned principle called

construction: If a state of affairs [*U*p] obtains, then the existence of universal U helps ground [*U*p]’s obtaining.

Raven argues in favour of construction based on two Armstrongian accounts concerning the nature of instantiation. Under both accounts, universals and particulars contribute to *construct* states of affairs, such as instantiations, and they do so by either *constitution* or *overlap*. Under the first account, particulars and universals constitute states of affairs, where constitution is intended as a form of non-mereological composition (Raven, 2022, p. 4; Armstrong, 1989, 1997):

account 1: constitution: States of affairs are constituted by universals and particulars, where constitution is a form of non-mereological composition.

**Fig. 1:**
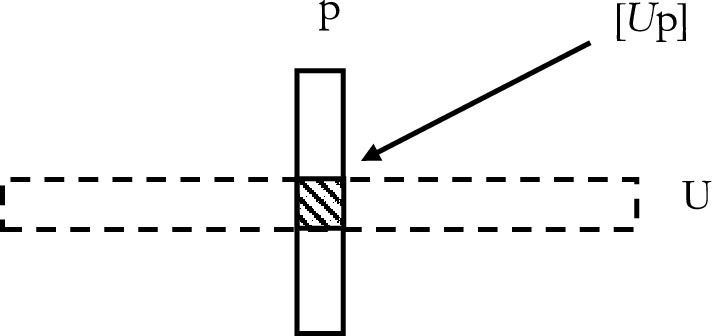
Account 2. States of affairs are the mereological product of universals and particulars

Under the second account, universals construct states of affairs by *overlapping* with particulars (Armstrong, 2004a, 2004b). Accordingly, the state of affairs that p instantiates U is the overlap (Raven, 2022, p. 5), of p and U (Fig. 1).

account 2: overlap: States of affairs are the overlap between universals and particulars.

In Raven’s text, it is possible to identify three distinct arguments to the effect that either view implies construction. We shall now consider them in turn.

### The argument from constitution

A first argument builds on account 1. Raven remarks that there is a sort of asymmetric counterfactual dependence in play in this case: a state of affairs such as [*U*p] can obtain only if U exists. But the reverse is not the case: U can exist without [*U*p]’s obtaining. One might take this as a reason for the metaphysical priority of U over [*U*p]. However, Raven himself discards this as an inconclusive argument, remarking that this kind of counterfactual dependence “won’t in general support priority” (Raven, 2022, p. 5). He is right, for counterexamples abound. Supposing for instance that numbers exist necessarily, my existence would be asymmetrically counterfactually dependent on, though arguably not metaphysically derivative from, that of, say, number 42. So much for this first argument from counterfactual dependence on which, admittedly, Raven himself seems not to put too much weight.

### The argument from overlap

A second argument builds on account 2 specifically. Recall that under such an account, a state of affairs is the overlap of universals and particulars. Raven takes this as evidence for the priority of universals over states of affairs, because, he suggests,

Overlap also seems to imply priority. The existence of the overlapping things is prior to the existence of their overlap, but not vice versa. (Raven, 2022, p. 5)

As such, this argument relies on an intriguing principle bridging grounding to overlap, according to which

priority of overlap: If *x* overlaps *y* and *z* is their overlap, the existence of *x* and *y* helps ground that of *z*.^3^

This principle is interesting, and worth a closer inspection. We notice here that account 2, and consequently priority of overlap, can be understood in two ways, depending on how we interpret ‘overlap’ and ‘part’. On a first interpretation, ‘overlap’ and ‘part’ maintain their mereological reading. On a second interpretation, they are to be understood in a non-mereological way—more on this later.^4^ Let us see how priority of overlap fares in both readings, starting with the mereological one.

In its mereological reading, the principle is prone to straightforward counterexamples. First, notice that everything overlaps itself. In that case, the overlap is the thing itself. Now, if priority of overlap is assumed, this entails that for any *x*, the existence of *x* helps ground itself—a ubiquitous violation of Raven’s asymmetry of grounding. Requiring the overlapping things to be numerically different from each other would not be enough. To see this, notice that everything overlaps any of its proper parts. In that case, the proper part itself is their overlap. If priority of overlap is assumed, this again entails a ubiquitous violation of asymmetry: for anything that is proper part of something, the existence of that thing helps ground itself. At this point one might try to rescue the principle by restricting it to cases in which the overlap is numerically different from the overlapping things. But, again, the resulting principle would be prone to plausible counterexamples. If there is a mereological sum of, say, Cleopatra and Caesar, as well as Cleopatra and Mark Antony, Cleopatra is going to be their overlap. But, plausibly, such arbitrary sums are not metaphysically prior to Cleopatra. A similar case does not involve arbitrary sums, but rather arbitrary partitions. Consider my body and my brain. There are plenty of ways of dividing my body into parts such that my brain is going to be their overlap. This implies the strange claim that the existence of my brain is partly grounded in the existence of any such parts. At this point, one might try to restrict further the principle in order to exclude cases in which arbitrary sums or arbitrary partitions are involved. But then again, plausible counterexamples could be found. Consider for example the case of two adjacent houses. In this case, the overlap is going to be the shared wall, whose existence is going to be partially grounded in that of each house. A similar case is that of two overlapping piles of sand, whose existence will help ground that of the shared grains of sand. Both are cases in which a sum, such as a pile of sand, is prior to its parts, such as some grains of sand. More generally, any application of priority of overlap will entail a case in which a whole is prior to at least one of its parts. Notice that in case arbitrary sums are allowed, priority of overlap would entail that any part would be metaphysically derivative with respect to any whole, with one odd exception, i.e. the universe (for there is no *x* such that *x* is distinct from the universe, overlaps with the universe, and such that their overlap is distinct from *x*). Now, the priority of wholes over parts has of course been defended, for example and oddly enough, exactly in the case of the universe (Schaffer, 2010). However, the idea that wholes are prior to their parts sits particularly badly with someone who, like Raven, takes “the existence of the constructing items to be prior to the existence of what they construct” (Raven, 2022, p. 5).

Let us now pass to the non-mereological reading. Remember how Raven introduces account 2. In his words (Raven, 2022, p. 5),

On the second paradigm, states of affairs are constructed from universals by overlapping them (Baxter, 2001; Armstrong, 2004a, 2004b). Universals and particulars can be partially identical. They are partially identical if they overlap. And they overlap if they share a part. This part is their overlap.

In order to give a non-mereological reading to this account, terms such as ‘overlap’ and ‘part’ should here be understood non-mereologically.^5^ How this can be properly done will depend on the particular account of partial identity that is adopted. Consider for example Baxter’s account. In it, entities have aspects, and such aspects are numerically identical to the entities that have them. Instantiation is then conceived as a universal and a particular sharing an aspect. For example, suppose that Socrates is wise. Socrates is then going to have an aspect, i.e. Socrates insofar as he is wise, and wisdom is going to have an aspect, i.e. wisdom insofar as Socrates has it, and these are the same aspect (Baxter, 2001, p. 454). Consequently, ‘parthood’ might be intended as the relation of numerical identity between an entity and any of its aspects, and ‘overlap’ as the fact that some aspects of different entities are in fact the same.

The fate of priority of overlap under this non-mereological reading will depend on the specific non-mereological account that is adopted. Of the two mentioned by Raven, Armstrong’s account (2004a) is the one that seems to sit worse with what Raven has in mind. That’s because Armstrong’s account makes instantiation necessary (2004a, p. 45), while Raven allows it to be contingent (Raven, 2022, p. 5). On the other hand, if Baxter’s account is adopted, priority of overlap would entail the priority of a thing over its aspects. However, as explained before, Baxter takes a thing and its aspects to be numerically identical (Baxter, 2001, p. 449) while, arguably, if *x* is prior to *y*, then *x* and *y* should not be numerically identical. So, this non-mereological reading, while opening a new possible line of research, is still insufficient to properly warrant the assumption that “overlap seems to imply priority” (Raven, 2022, p. 5).

### The general argument

Raven provides us with a third argument in favour of the priority of universals over their instantiations. It is also arguably the argument on which he counts the most. The argument builds on the fact that under both account 1 and 2, there is a sense in which universals construct, either mereologically or non-mereologically, the states of affairs that are their instantiations. Raven adds—and he repeats this twice—that

An item cannot be constructed by what it helps to construct. This supports taking the existence of the constructing items to be prior to the existence of what they construct (Raven, 2022, p. 5)

To unpack a bit, Raven is here suggesting that any constructing relation, be it mereological or non-mereological, obeys asymmetry:

asymmetry of construction: (AC) If *x* helps construct *y*, then it is not the case that *y* helps construct *x*.

Moreover, he is claiming that this kind of asymmetry, characterizing the relation of construction, supports the priority of the constructing items over what they construct, namely the following principle:

priority of constructing items: (PCI) If *x* helps construct *y*, then the existence of *x* is prior to the existence of *y*

Why does Raven take AC to support PCI? Let us try to formulate an argument to this effect. It is evident that at least one premise is here left implicit, linking the asymmetry of construction to the priority of the constructing items over what they construct. Why does Raven take this alleged asymmetry to support priority? It can’t be that the simple asymmetry of a relation could make it imply metaphysical priority. Plenty of asymmetric relations are not correlated with priority. This is recognized by Raven himself, who, as noted above, concedes that no claim concerning metaphysical priority can be legitimately derived from an *asymmetric* case of counterfactual dependence. If any concrete example is required here, one needs just to think that ‘being taller than’ and ‘being colder than’ are asymmetric relations, but they arguably do not imply corresponding priority claims. So, Raven must think that there is something specific to the asymmetry of the relation of *construction* which makes it systematically correlated with priority. However, the crucial task of filling this argumentative gap is unfortunately left to the reader.

In trying to fill this gap, we should keep in mind that establishing a correlation between construction and priority would still not be enough for Raven’s aims. To see this, let us grant, for the sake of the argument, that there is such a correlation. Now, as in any case in which two correlated asymmetric relations are involved, the question will still remain to be answered: which direction is correlated with which? Should what constructs be more fundamental? Or should what is constructed be more fundamental? The simple correlation between construction and priority does not push towards any of these two options. An appeal to the existing literature on this matter would not be of much help here. For in both mereological and non-mereological cases of construction, we find authors defending the metaphysical priority of what is constructed over what constructs. For example, the already mentioned Schaffer (2010) argues that the universe, which is mereologically constructed by any concrete entity, is metaphysically prior to what constructs it. And the late Armstrong himself (2012) argues that states of affairs are metaphysically prior to what non-mereologically constructs them. So, in order to succeed, Raven would have to provide us with sufficient reasons to prove both Schaffer and the late Armstrong wrong.

Alternatively, Raven might try to fill this gap by assuming that the notion of priority is *built in* his notion of construction, so that it is in the very the notion of construction the fact that it implies priority.^6^ Following this idea, let’s distinguish two notions of construction, general *vs* strict:

1. *x* is *generally constructed* by *y* iff *x* is either mereologically on non-mereologically constituted by *y,*
2. *x* is *strictly constructed* by *y* iff *x* is *generally constructed* by *y* and *y* is prior to *x*.^7^

In other words, the alternative we are here discussing is that Raven is operating with a notion of *strict construction*. This would have at least two consequences for our discussion. First, our counterexamples would be discarded. Take for example Schaffer’s universe. We mention it as a case of an entity that is prior to, and yet constructed out of, its parts. But if construction is strict construction, this can’t be the case. Since the universe is prior to its parts, it would not be constructed, in the relevant sense, out of its parts. Second, we have spent much time in discussing Raven’s justification for the claim that construction implies priority. But if priority is built in the relevant notion of construction, this task is arguably considerably easier.

Let us take a closer look on this alternative. Remember that Raven says that (i) states of affairs are constructed out of universals, and that (ii) construction implies priority. On the one hand, taking construction in the strict sense would make (ii) easier to prove, but would at the same time make (i) to be in dire need for a justification. If construction is intended in the strict sense, what evidence do we have that states of affairs are constructed by universals in the strict sense? On the other hand, as discussed above, taking construction in the general sense, as we take Raven to be doing, would make (i) less controversial, but would at the same time make (ii) to be in dire need for a justification.

Finally, we notice that so far we have worked under the hypothesis that (i) and (ii) are to be somehow justified. However, it might be the case that Raven’s aims are much more limited, in that he is working with the strict notion and *assumes* that states of affairs are constructed by universals in this sense. In this case, Raven would not be arguing in favour of either (i) or (ii), but simply exploring the implications of their conjunction.^8^ Here, we simply observe that this reading would substantially reduce the scope of Raven’s paper, since it would only target those Aristotelians who assume that universals construct states of affairs and that metaphysical priority is built in the relevant notion of construction.

## Raven’s argument requires the transitivity of grounding

We are now going to argue that, on closer inspection, Raven’s argument requires the transitivity of grounding. To see this, we go back to Raven’s way of arguing that immanence and construction entail a violation of asymmetry. There, a problem was that while construction concerns specific universals and states of affairs, immanence concerns generic states of affairs: the existence of a universal is grounded in *some* instantiation of it. The problem is allegedly solved by making use of existential grounding, whereby existential generalizations are grounded in each of their instances. But how does existential grounding solve the issue here, precisely? Raven writes (2022, p. 6):

We use a scenario in which p instantiates U (…). Since p instantiates U, U exists. By immanence, some instantiation of U helps ground U’s existence. And by e. grounding, p’s instantiating U helps ground U’s existence.

Raven’s point here is quick, and a little bit of formalism will help in inspecting it. With $<$ for partial metaphysical grounding, ‘E*x*’ for ‘*x* exists’, we can render formally both.

immanence: If U exists, some instantiation of it helps ground its existence
  $$E{\text{U}} \to \exists x\left( {Ux} \right) < E{\text{U}}$$

and

existential grounding: If *U*p, then [*U*p] helps ground [something’s being *U*]
  $$U{{p}} \to U{{p}} < \exists {\text{x}}\left( {U{\text{x}}} \right)$$

Formally speaking, hence, Raven’s argument is:
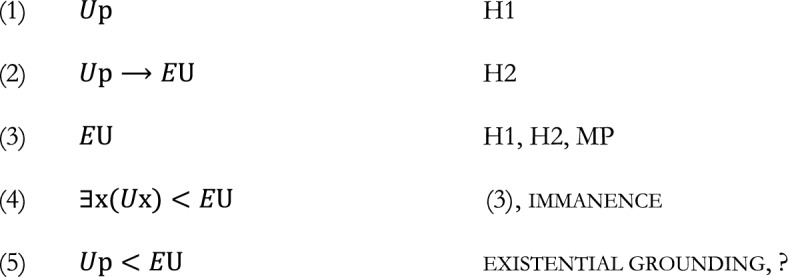


A crucial question is how to get to (5). We know that (5) is supposed to follow from an application of existential grounding. However, to which line was it applied? It can’t be line (4), because the antecedent of existential grounding is of the logical form $U\mathrm{p}$, and (4) does not have this logical form. It might be line (1), which has the right logical form. But if existential grounding is applied to (1), we don’t get (5), but rather
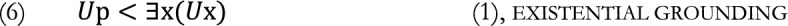


Now, there is an obvious way of getting from (6) and (4), which are already established, to the desired (5), namely by assuming the *transitivity of partial grounding*:
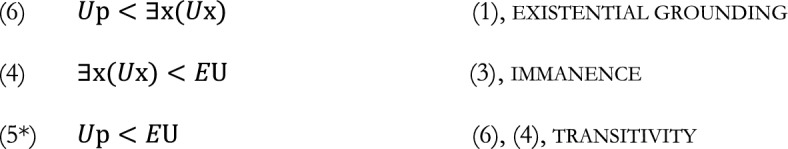


But this comes at a cost: Raven’s version of the argument relies on the transitivity of ground and therefore loses its alleged advantage over its predecessors.

Are there any other ways to get to the desired (5)? Here is one. We assume that (A) for a universal to exist is for it to be exemplified, and that (B) the terms of grounding are substitutable with factually equivalent ones *salva veritate*. Under such assumptions, in (6), we can replace the grounded state of affairs $[\exists {\text{x}}(Ux)]$ with the state of affairs that U exists. However, each of these claims comes with significant problems. (B) is not totally implausible, but certainly controversial given the hyperintensionality of grounding (Berto & Nolan, 2021; Duncan et al., 2017). (A) has a distinct Aristotelian flavour. It seems indeed an alternative way of capturing the Aristotelian view. However, it would also make the argument somehow redundant and certainly more controversial. Somehow redundant, in that it would employ two different ways of capturing the Aristotelian view. Certainly more controversial, in that it commits the Aristotelian view to ontological pluralism (for it claims that for universals to exist is to be exemplified, whereas, arguably, for other things to exist is not for them to be exemplified). In light of the fact that these two assumptions are significantly controversial, and that there is absolutely no trace of them in Raven’s paper, we conclude that the most sensible way to rescue the argument is indeed to assume the transitivity of partial grounding.

One could try to improve Raven's argument by assuming an alternative reading of the immanence principle, namely^9^

immanence*: If U exists, some instantiation of it helps ground its existence
  $$E{\text{U}} \to \exists {\text{x}}(U{\text{x}} < E{\text{U}})$$

This is slightly different from our reading of immanence for in immanence * the quantifier takes a wider scope.

Be it as it may, it is crucial to note that also this alternative reading would not fill the argumentative gap we are discussing here. Indeed, going back to the relevant passage of the argument, and substituting immanence * for immanence, we get: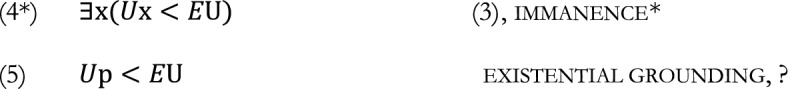 It still remains unclear how we could get from (4*) to the desired (5) by means of existential grounding. Notice that (5) cannot be derived from (4*) in virtue of the rule of existential elimination. First, this would result in an argument different from that of Raven, since it makes use of the elimination rule, and it does not make use of Raven’s existential grounding. Second, and crucially, this hypothesis would make the argument invalid, since *p* already occurs in the derivation, at line 1.

## A new argument against Aristotelian universals (this time without transitivity)

We have just shown that, in order to be valid, Raven’s argument requires the transitivity of ground. We don’t take this to be problematic, insofar as we believe grounding to obey transitivity. Still, in what follows, we provide a new argument against Aristotelian universals which successfully avoids any commitment to transitivity, thus vindicating Raven’s intuition that this could be done, and thus satisfying those who, unlike us, are skeptical about transitivity.

The argument builds on a different way of capturing immanence, or the Aristotelian view. In general terms, this is the view according to which universals depend on their instantiations. But on which of their instantiations do they depend? Raven’s immanence principle gives a somehow minimalist answer to this question. A universal depends on *at least some* of its instances, as testified by the presence of the existential quantifier in the antecedent:

immanence: If U exists, some instantiation of it helps ground its existence
  $$E{\text{U}} \to \exists {\text{x}}\left( {U{\text{x}}} \right) < E{\text{U}}$$

The same minimalist rendering of the Aristotelian view is also at work in the preceding versions of the ground-theoretic argument against Aristotelian universals (Alvarado, 2020; Costa, 2021). Still, the Aristotelian view is plausibly intended to also imply the claim that a universal depends on *any* of its instances, and not on some of them only. After all, to use a somehow colorful expression, *any* of the instances of the universal would be sufficient for bringing the universal to existence. Yet in other words, under the Aristotelian view, it would be difficult to find an instantiation of a universal that would not imply, and ground, the existence of the universal itself.^10^ This further intuition can be captured by the following, stronger, principle of immanence, call it

strong immanence: Any instantiation of universal U helps ground its existence
  $$\forall {\text{x}}(U{\text{x}} \to (U{\text{x}} < E{\text{U}}))$$

It is not difficult to show that, under the assumption that the existence of a universal implies its being instantiated, strong immanence entails the already discussed immanence *.^11^ It is also interesting to note that immanence and strong immanence are two significantly different ways of capturing the Aristotelian view, as explained above. Their difference deserves to be properly appreciated.

The reader might also have noticed a formal similarity between our strong immanence and the quantified version of the principle previously called existential grounding:

existential grounding (quantified version): Any instantiation of a universal helps ground the fact that it is instantiated

$$\forall {\text{x}}\left( {U{\text{x}} \to U{\text{x}} < \exists {\text{y}}\left( {U{\text{y}}} \right)} \right)$$

Despite their similarity, the two principles are crucially different. It is worthwhile to contrast them here. As a matter of fact, only the former delivers a version of Aristotelianism, insofar as the former, and not the latter, concerns the existence of universals and its ground. If we take redness as an example, existential grounding concerns the ground of the fact that something is red, while strong immanence concerns the existence of redness itself. As such, existential grounding, but not strong immanence, could even be accepted by a Platonist, who denies that the existence of universals is grounded in their exemplifications.

Once strong immanence is on the table, a straightforward argument against the Aristotelian view, as formulated here, can be provided. Like Raven’s and the preceding versions of the ground-theoretic argument, it builds on the further assumptions that the existence of a universal grounds any of its instantiations (i.e. what Raven calls construction), and that the instantiation of a universal entails its existence (H2 above).

construction: If a state of affairs [*U*p] obtains, then the existence of universal *U* helps ground [*U*p]’s obtaining.

$$U{\text{p}} \to E{\text{U}} < U{\text{p}}$$

The argument runs as follows:
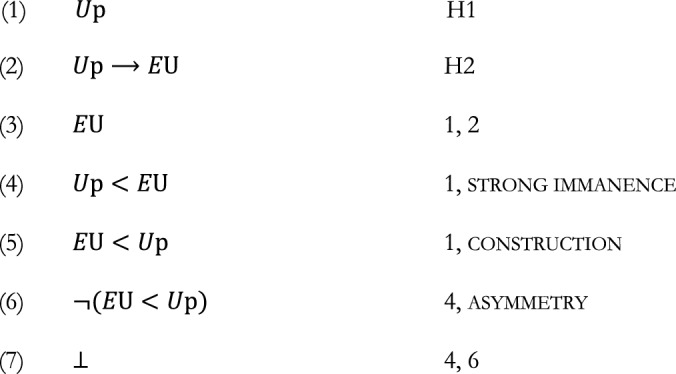


In other words, in the presence of construction and asymmetry, the Aristotelian view as captured by strong immanence is to be rejected. In addition, since the existence of a universal implies its being instantiated, the same argument allows us to conclude that no universal exists (provided that strong immanence, construction, and asymmetry are still accepted).

It's not hard to see why this argument differs from Raven’s (as well as from its predecessors in Alvarado, 2020 and Costa, 2021), in that it doesn’t make use of some of Raven’s assumptions (most notably immanence and existential grounding) and at the same time makes use of assumptions Raven doesn’t make (i.e. strong immanence).

## Conclusion

In assessing Raven's proposal, we yielded some significant contributions to the debate on Aristotelian universals. Our analysis, besides shedding light on the limitations of Raven's iteration of the argument, has introduced a novel ground-theoretic rendering of the Aristotelian view, i.e. strong immanence. In light of it and considering that asymmetry is hardly disputable as a principle concerning grounding, it seems that a theory of universals can only succeed if at least one of strong immanence and construction is rejected. So, a realist should either put into question that universals are grounded in their instances or avoid the idea that states of affairs are constructed out from universals, or in any case grounded in facts involving them.

## Footnotes

1 More precisely, Raven assumes that grounding obeys asymmetry and existential grounding (see below) but does not assume it obeys transitivity. He also assumes the standard distinction between partial and full ground (Raven 2022, p. 2).

2 Throughout the paper, we use ‘U’ for indicating the universal corresponding to the predicate ‘*U*’. Moreover, [φ] will indicate the state of affairs that φ.

3 An anonymous referee suggested that here Raven might not be endorsing priority of overlap, but a more local principle instead, which concerns the case of a universal and a particular being partially identical. However, Raven seems to explicitly assume that priority of overlap when he writes: “This supports taking the existence of the constructing items to be prior to the existence of what they construct. In general, the overlap exists in virtue of the existence of the overlapping things. In our case, a state of affairs [*U*p] is the overlap of particular p and universal U” (Raven, 2022, p. 5). Here, the local case (“our case”) of partial identity is contrasted with the general case of overlap ("in general"), and the priority of the specific case is inferred from the priority of the general case.

4 We thank an anonymous referee for pushing us to consider this second reading.

5 Incidentally, we notice some facts in favour and against this non-mereological reading. In favour, the fact that Raven mentions Baxter and Armstrong, who clarify that their account of partial identity is non-mereological (Armstrong 2004b, p. 145, Baxter, 2001, p. 463). Against, the fact that Raven uses seemingly mereological terms such as ‘overlap’ and ‘part’ without making the reader aware that they are intended in a non-mereological sense. Moreover, Raven makes the effort of clarifying that ‘constitution’ in account 1 is to be intended non-mereologically (Raven 2022, p. 5). One would therefore expect a similar clarification when the risk of confusion is higher, namely when presenting account 2.

6 Though we don’t take this reading, suggested by a reviewer, to reflect Raven’s original thoughts, as explained below, we agree that it is not totally without textual evidence either. For example, Raven talks here about a “priority-laden notion of construction” (Raven 2022, p. 5).

7 We provide these two definitions for ease of clarity. However, what follows does not depend on the claim that these are defined notions rather than primitive ones. As long as their meaning is clear, what we say below should follow anyway.

8 We thank an anonymous referee for suggesting this possible reading. We lean towards the previous readings in light of the fact that Raven takes up a substantial part of his paper arguing in favour of (ii).

9 We are grateful to Martin Glazier and an anonymous reviewer for drawing our attention towards this reading.

10 We are grateful to Joshua Babic for drawing our attention on this important difference.

11 Suppose that U exists. Then there is some x that instantiates U, since universals exist only if they are instantiated, and therefore there is some instance x of U such that [*U*x] helps ground the existence of U, by strong immanence.
